# Book Reviews

**Published:** 1900-03

**Authors:** 


THE SURGICAL DISEASES OF THE GENITO-URINARY TRACT, VENEREAL AND SEXUAL DISEASES. A Text Book for Students and Practitioners. By G. FRANK LYDSTON, M. D., Professor of the Surgical Diseases of the Genito-Urinary Organs and Syphilology in the Medical Department of the State University of Illinois; Professor of Criminal Antropology in the Kent College of Law; Surgeon-in-Chief of the Genito-Urinary Department of the West-Side Dispensary; Fellow of the Chicago Academy of Medicine; Fellow of the American Academy of Political and Social Science; Delegate from the United States to the International Congress for the Prevention of Syphilis and the Venereal Diseases, held at Brussels, etc. Illustrated with 233 engravings. Pages xvi.–1024. Philadelphia, New York and Chicago: The F. A. Davis Co. 1899.

When a lordly and egotistical gentleman with an M. D. hooked on the tail end of his name gets a job as lecturer in a medical college, his next step is to write a book, and of a truth his work tells just what he has read in other books and as a result, his literary contribution is one of those frappéd works of art which produce cross-eyes, and brain fag, leaving unpleasant memories in its trail. That is why medical literature to-day is a sort of a junk-bag of rewritten works and rehashed experiences; and that is why, when a man with a full and comprehensive knowledge of any special branch of medicine, sits down and tells all he knows of his subject, and gives his own views, as well as the views of others, in a book which is well-written, intelligently builded, and tells things which are not generally known, he has done a creditable act and deserves commendation for it. That is why the practising physician should feel under personal obligation to Dr. Lydston. The publisher is a sort of intermediate means to a general end, yet the publishers of this book should be credited with exceptional good taste.

The author does not apologise for writing his book and he does not hesitate to say so either, which is a good sign at the outset. When a man apologises for writing a book, it means that he needs a brace; that he has a shaky confidence in his work. In this case, neither apology or explanation is needed. The book is quite able to stand on its merits, and stand firmly to. Besides, compared subject for subject, it out-classes most of the existing volumes in genito-urinary subjects and makes them look like primers; and, of a truth, many of them have that quaint a-b ab flavor of our careless childhood days.

There are ten parts in the treatise which Lydston has given us, and each one is a gem, no matter from whichever view point it is inspected. In part one Dr. Lydston very wisely takes up the general principles of genito-urinary, sexual and venereal pathology and therapeutics, and as wisely leaves it there, without trailing it through to the last chapter. Then comes nonvenereal diseases, diseases of the urethra and gonorrhea, chancroid, bubo and their complications, syphilis, diseases affecting sexual physiology, diseases of the urinary bladder, surgical affections of the kidney and ureter, and diseases of the testes and spermatic cord.

A strong claim of the book lies in the fact that each of these subjects is handled in a masterly fashion and completed in the section devoted to it. The operation procedures are presented in what might very properly be called a distinctively American manner; that is by adequately describing the methods advised, and going into detail as regards treatment. This is in strong contrast to some recent foreign works on genito-urinary subjects and monographic contributions on special branches, as for instance the still new publication, Die Krankheiten der Prostata, by Dr. A. Van Frisch, Professor of Surgery in Vienna, who bows low before the shrine of Battini and, giving his opinion of radical measures in prostatectomy without supporting them, calmly neglects to give any intelligible description of operative procedures. In his work, Dr. Lydston has made himself so clear as regards operative work that, after a careful and conscientious study of the book, any senior medical student of the normal type should feel a deep sense of confidence in his ability to make a creditable showing in doing much of the work laid down.

This book gives brilliant promise of further work in this field by the distinguished author, and its later issues, for assuredly there will be other editions, will keep it up to the standard of a masterpiece of genito-urinary literature. The author very gracefully and most appropriately dedicates his work to Dr. Fessenden N. Otis.

round or oval;" (2) "bacilli, which are rod-shaped cells," and (3) "bacteria, which may be rod-shaped; . . . . also oval."

I read the book, but I did it as a duty and some duties are so disagreeable that they have a leathery taste in one's mouth. As I have said the printing, the binding and the pictures are good. As for the book as a book, well, read that McGuire story again!

N. W. W.

---

The International Text-Book of Surgery. By American and British Authors. Edited by J. Collins Warren, M. D., LL. D., Professor of Surgery in Harvard Medical School; Surgeon to the Massachusetts General Hospital; and A. Pearce Gould, M. S., F. R. C. S., Surgeon to Middlesex Hospital, and Teacher of Operative Surgery at Middlesex Hospital Medical School; Member of the Court of Examiners of the Royal College of Surgeons, England. Volume I. General and Operative Surgery. With 458 illustrations in the text, and nine full-page plates in colors. Philadelphia: W. B. Saunders. 1899. [Price, $5.00.]

The wealth of surgical literature that has been added to so much in recent years, is greatly increased by the publication of this treatise. This first volume, at all events, gives token of the fact that a splendid contribution is making to our surgical libraries.

The field covered by this volume, general and operative surgery, affords an opportunity for the editors to command the pens of some of the ablest and most practical surgeons. For example, they have laid under contribution such men as Harold C. Ernst, who writes of surgical bacteriology; Richard C. Cabot, who contributes a chapter on the surgical pathology of the blood; George Ryerson Fowler, whose chapter on wounds and contusions, burns and scalds, effects of lightning, shock, fat embolism, and repair of special tissues, is most practical; Weller Van Hook, with two chapters, one on constitutional reactions to wounds and their infections, and another on hydrophobia, anthrax, glands, etc., which are instructive; Walter George Spencer, who writes on gangrene with precision; I. H. Cameron, on surgical tuberculosis; and three chapters by the senior editor. These comprise the contributors to general surgery in this volume.

In operative surgery the writers are McBurney, Warren Da Costa, Gay, Pfaff and Burns, Bland Sutton, Pilcher and Warbasse, Makins, Cheyne, Willard, Rushton Parker, Bradford and Lothrop, Monks, Tiffany, Golding, Bird, Richardson, Burrill, and the late John B. Hamilton.

McBurney's chapter, on the technic of aseptic surgery, is a complete exposition of the subject by a practical surgeon and is clearly, even fascinatingly, presented. It should be carefully studied by every young practitioner who essays to do the surgery that comes within his grasp. A complement to this chapter is one on Anesthetics and surgical anesthesia, by George W. Gay, Franz Pfaff and T. G. A. Burns. The two important preliminaries to all good surgery are skilful administration of the anesthetic, and the intelligent, thorough application of the principles of asepsis.

These are the two contributions of the XIXth. century to the surgical art that have done more than all others in all time to make surgery a humane, life-saving, and health restoring art. Gay and his colleagues have risen to the occasion in their contribution, and have given us a practical chapter on anesthesia and anesthetics.

Bone surgery is considered in several chapters--one on fractures, by Lewis S. Pilcher; one on injuries of the joints and dislocations, by George Henry Makins; one on dislocations of the hip, by J. Collins Warren; one on diseases of the bones by Watson Cheyne; one on diseases of the joints, by De Forest Willard, and one on diseases of special joints (orthopedic surgery) by Rushton Parker. Besides these there are other chapters that involve bone surgery to a greater or less extent, such as one on congenital dislocation of the hip, flat-foot and club-foot, by E. H. Bradford and Howard Lothrop; one on cranial surgery, by L. McLane Tiffany; and one on surgery of the spine, by Golding Bird.

Other chapters requiring special mention are one on hyperemia, inflammation, etc., and one on suppuration, abscess, etc., by J. Collins Warren; one on minor surgery, by John Chalmers DaCosta; one on tumors, by J. Bland Sutton; and one on surgery of the peripheral nerves, by Maurice H. Richardson.

Several other important topics are ably presented by competent men. The scope and method of the treatise are commendable, and the work when completed will constitute a noteworthy addition to the surgeon's library.

LOVE AND ITS AFFINITIES. By GEORGE F. BUTLER, M. D, Professor of Materia Medica and Clinical Medicine in the College of Physicians and Surgeons, Medical Department of the University of Illinois. Author of "Materia, Therapeutics and Pharmacology," etc. Beautiful octavo volume, cloth, gilt top, 134 pages, with photogravure frontispiece, "Cupids Sharpening Their Arrows," by Raphael Mengs. Chicago: G. P. Engelhard & Co., Publishers.

The affinities of love which Dr. Butler discusses are lust and religion. To the thoughtful man or woman accustomed to studying the motives of mankind, the association is not startling. In the last analysis motive resolves itself into one or the other of the fundamental impulses, self-preservation or the preservation of the species; and while at first it seems a far cry from the baseness of the depraved sexual instinct to the sublime heights of religion, the relation between the two is not so distant. Both have their origin in the same passion.

Phallicism, the almost universal sex-worship of primitive peoples, shows the alliance between religion and the sexual instinct, an alliance seen, but scarcely ever appreciated, in the greater religious receptivity in early life when the sexual powers are maturing.

Permit the reviewer to suggest for the greater religious devotion of women a reason which seems not to have occurred to the author. Love, the gratification of the sexual instinct, demands of woman, has demanded of the brute mother for long ages before man appeared on the globe, self-sacrifice. For woman self-sacrifice is a corrolary of love. Does she not turn to the church because the self-abnegation, which all religion enjoins, affords an outlet to the altruistic instinct denied its natural expression in motherhood?

Of those whose work does or should acquaint them with the elemental passions of mankind, the physician, who sees too often the grosser side of the sexual instinct, needs to have shown him, as Dr. Butler has done, the reverse of the picture, which deals with love in its higher forms. Too prone is he to regard the base and animal as the only manifestations of the all-pervading and all-compelling passion. On the other hand, there are those by whom far more knowledge of this fundamental impulse and its affinities might well be possessed—the professional teacher and the preacher. The one has the guiding of the young through the critical periods of youth and early manhood, maidenhood and young womanhood; the other, engaged with the ideals of life is not infrequently unacquainted with the real man and the instincts and passions which make him what he is. Let the physician read that he may not wholly lose faith in human nature; the priest and teacher, that they may understand more thoroughly how closely allied in man are the worst and the best.

Dr. Butler has avoided the pathological. Throughout the volume he has quoted widely from the literature of all ages in proof and illustration of his statements, and while laying no claim to originality, he presents a difficult subject with delicacy and beauty.

---

A TEXT-BOOK OF THE PRACTICE OF MEDICINE. By JAMES M. ANDERS, M. D., Ph. D,, LL. D., Professor of the Practice of Medicine and of Clinical Medicine in the Medico-Chirurgical College, Philadelphia; Attending Physician to the Medico-Chirurgical and Samaritan Hospitals, Philadelphia, etc. Octavo, pp 1292. Illustrated. Third edition, revised. Philadelphia: W. B. Saunders, 925 Walnut street. 1899.

It is a genuine pleasure to present repeated notices of a book like this one, for it means that it is appreciated by physicians and students of medicine. Especially does it mean in this instance that teachers have recognised its superior merits, have recommended to their classes, and have adopted it in their schools as a leading text-book.

In former reviews we have discussed the general make-up of the treatise, and many of its salient features; in this we need only speak of changes. This edition exhibits the results of careful revision. The first part, entirely devoted to the consideration of infectious diseases, has felt the effect of the revisers hand in a marked degree, owing to the fact that our knowledge of these diseases has advanced considerably of late. It is important—most important—that text-books on practice should keep well to the fore in this department, and should note every advancement in a group of diseases that so intimately relates not only to public health, but to private welfare. This author has demonstrated his appreciation of this assertion, by bringing his book forward to the immediate present in its consideration of infectious diseases.

Again has the author kept pace with events through the introduction of a group of new subjects, the rewriting of several topics of great importance, and the extensive review of many articles; for example, typhoid and yellow fevers, lobar pneumonia, tuberculosis, etc., etc., all of which combine to present strong evidence of the high value of the treatise.

The book is essentially clinical in character, as might be anticipated when the eminence of the author as a clinical teacher and observer is recalled. This distinctly adds to the value of the work as a text-book and especially does it enhance its quality as a reference volume for younger physicians and family doctors.

Anders has had the good fortune to group many excellent qualities in his book, one of the most important being the manner of its arrangement. It differs from most others in its make-up and is excelled by none in the fashion of its typography, or in the division and subdivision of its subjects.

Finally, this treatise is commended as one of the strongest text-books on the practice of medicine yet offered to the profession or undergraduate.

GENERAL AND LOCAL ANESTHESIA. By AIME PAUL HEINECK, M. D, Clinical Instructor in Genito-Urinary Diseases, College of Physicians and Surgeons, Chicago; Clinical Instructor in Gynecology, Chicago Clinical School; Clinical Instructor in Surgery, Northwestern University Woman's Medical College. 124 pages. Chicago: G. P. Engelhard & Co., Publishers, 358-362 Dearborn street.

In this eminently practical monograph the author has succeeded in detailing very plainly the rules pertaining to the giving of general and local anesthetics. Ether and chloroform constitute the first topic, the selection of the anesthetic, precautions before operation, symptoms during and after the anesthetic state. The latter half of the book is devoted to the various ways of producing local anesthesia by refrigeration, by cocaine and by Schleich's infiltration method. Cocaine especially is dwelt upon; its use in the various specialties and the manner of injection in distinct operation; for example, circumcision, are given in full.

The monograph will prove valuable to the student and surgeon alike, for the English is simple, all theories and historical data are omitted and only such details as experience has proved important, are given. The book is printed and bound attractively.

A LABORATORY MANUAL OF PHYSIOLOGICAL CHEMISTRY. By ELBERT W. ROCKWOOD, B. S., M. D., Professor of Chemistry and Toxicology in the University of Iowa. Illustrated with one colored plate and three plates of microscopic preparations, 5⅛ x 7¼ inches. Pages viii.—204. Philadelphia: The F. A. Davis Co., Publishers, 1914-16 Cherry St. 1899.

This book has been prepared with a view to aid the student in laboratory work, by imparting knowledge through the observation of the student himself. In addition to the directions for experimental work, brief explanation of facts are given. With some acquaintance with general chemistry and chemical manipulation, the student will obtain much aid from this brochure. The section devoted to the urine is especially to be commended and will be found a useful guide in conducting researches in diseases of the urinary tract.

